# Using multi-theory model to predict initiation and sustenance of small portion size consumption among college students

**DOI:** 10.15171/hpp.2016.22

**Published:** 2016-08-10

**Authors:** Manoj Sharma, Hannah Priest Catalano, Vinayak K. Nahar, Vimala Lingam, Paul Johnson, M. Allison Ford

**Affiliations:** ^1^Behavioral & Environmental Health, School of Public Health, Jackson State University, MS, USA; ^2^Public Health Studies, School of Health and Applied Human Sciences, University of North Carolina Wilmington, NC, USA; ^3^Department of Health, Physical Education, and Exercise Science, School of Allied Health Sciences, Lincoln Memorial University, Harrogate, TN, USA; ^4^Department of Health, Exercise Science & Recreation Management, School of Applied Sciences, University of Mississippi, MS, USA; ^5^Department of Management, School of Business Administration, University of Mississippi, MS, USA

**Keywords:** Obesity, Overweight, Portion size, Diet, Nutrition

## Abstract

**Background:** Consumption of large portion sizes is contributing to overweight and obesity.College students are a vulnerable group in this regard. The purpose of this study was to use multi-theory model (MTM) to predict initiation and sustenance of small portion size consumption in college students.

**Methods:** A total of 135 students at a large Southern US University completed a 35-item valid (face, content, and construct) and reliable (internally consistent) survey electronically in a cross-sectional design. The main outcome measures were intention to start eating small portion sizes and continuing to eat small portion sizes. Only those students who ate large portion sizes during the past 24 hours were included.

**Results:** Step wise multiple regression showed that initiation of small portion size consumption was explained by participatory dialogue (advantages outweighing disadvantages), behavioral confidence, age, and gender (adjusted R^2^ = 0.37, P < 0.001). Males were less likely to initiate small portion size consumption than females (β = -0.185, 95% CI = -0.71– -0.11). Regarding sustenance, emotional transformation, changes in social environment, and race were the significant predictors (adjusted R^2^ = 0.20, P < 0.001). Whites were less likely to sustain small portion size change than other races (β = -0.269, 95% CI = -0.97 – -0.26).

**Conclusion:** Based on this study’s findings, MTM appears to be a robust theoretical framework for predicting small portion size consumption behavior change. Interventions in this regard need to be designed.

## Introduction

Obesity is a public health crisis in the United States with more than one third (34.9%) of the adult population classified as obese.^1^ Obesity increases the risk for coronary heart disease, stroke, cancer, hypertension, type 2 diabetes, gallbladder disease, osteoarthritis, and sleep apnea.^2^ Food portion sizes are a major contributing factor to the obesity epidemic in the United States.^3^ Findings from research studies provide evidence that increases in food portion size have been directly proportional to increases in obesity rates.^4,5^ The concept of supersizing portion sizes is practiced almost all over the world with the greatest increases in the United States.^6^

Consumption of healthy portion sizes, and subsequently reduced caloric intake, is an essential strategy for obesity prevention.^7^ However, few educational interventions have been conducted to promote healthy portion size consumption.^8^ Portion size interventions have had mixed results, though none have explicitly applied a theoretical framework. However, one intervention^9^ applied the self-regulation construct from social cognitive theory.^10^ Most of the interventions targeted improving participants’ portion size estimation skills and increasing awareness and knowledge of appropriate portion sizes.^11-13^ College is a critical period in which individuals establish lifestyle behaviors, including dietary behavior, which impacts weight and long-term health outcomes.^14,15^ College students often have poor dietary habits, including low intake of fruits and vegetables, skipping meals, inadequate consumption of a variety of foods, consuming large portion sizes, snacking, frequent consumption of fast food, and high intake of high energy-dense foods.^14^ Only 3.8% of college students consume the recommended five servings of fruits and vegetables per day.^16^ Furthermore, several studies have reported substantial weight gain among college students throughout their college experience.^17,18^ Approximately one quarter (23.3%) of college students nationwide are overweight and an additional 16.3% of college students are obese. One approach to reducing obesity problem is to reduce portion sizes which will be the focus of this study. Sharma conceptualized the multi-theory model (MTM) to predict one-time and continuous health behaviors. The MTM proposes that health behavior change occurs through two components: initiation of the behavior change and sustenance of the behavior change. Existing health behavior theories have not considered this distinction with health behavior, which has often resulted in poor predictive power when operationalizing those constructs.^19^

**Figure 1:**
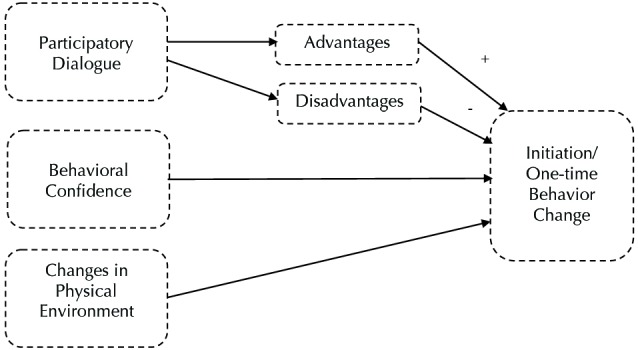
Constructs in initiation of health behavior change in multi-theory model (MTM) of health behavior change.

The MTM proposes that three constructs predict the initiation of behavior change, namely, participatory dialogue (in which advantages outweigh disadvantages), behavioral confidence, and changes in physical environment. Participatory dialogue is similar to the perceived benefits and perceived barriers constructs within the health belief model (HBM) and pros and cons within the trans-theoretical model (TTM).^20,21^ However, the construct of participatory dialogue is unique because it requires participation and mutual exploration, which is a process that Freire emphasized but is disregarded by the HBM and TTM.^22^ Behavioral confidence is derived from Ajzen’s ^23^ perceived behavioral control construct and Bandura’s^10^ self-efficacy construct. However, behavioral confidence is distinct from these constructs in that the target is on changing behavior in the future rather than at the present time.^19^ Thus, behavioral confidence is defined as how sure an individual is that he/she can perform a behavior change in the future. Furthermore, behavioral confidence acknowledges that an individual’s source of confidence is not exclusively internal. Thus, behavioral confidence may be derived from external sources such as important people in life, higher being, health educator, etc.^19^ The changes in physical environment construct only pertains to the physical environment and entails modifying the “obtainability, availability, accessibility, convenience, and readiness of resources.”^24^ Figure 1 presents constructs in initiation of health behavior change in MTM.

**Figure 2:**
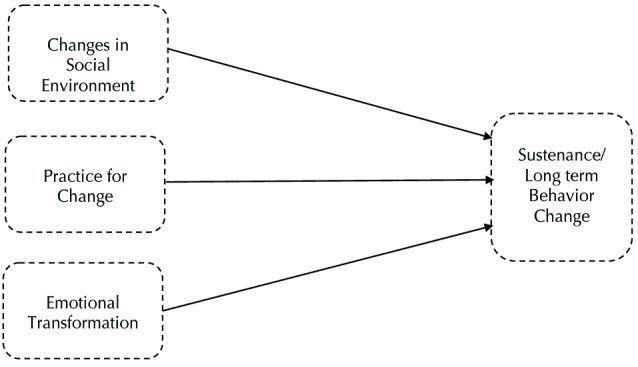
Constructs in sustenance of health behavior change in multi-theory model (MTM) of health behavior change.

Within the MTM, three constructs are posited to influence the sustenance of health behavior change or modification for health behavior change in the long-term. According to the MTM, emotional transformation, practice for change, and change in social environment explain and predict the sustenance of health behavior change.^19^ Emotional transformation involves collecting one’s own emotions and directing and transforming those emotions toward the health behavior change. Practice for change is derived from the praxis construct within Freire’s ^22^ adult education model, which refers to dynamic reflection and reflective behavior. The practice for change construct entails continually ruminating the health behavior change, integrating modifications to existing behavior change strategies, managing barriers, and maintaining focus on the health behavior change. The final MTM construct is change in social environment. The change in the social environment construct entails developing social support within the environment. A variety of professionals including health educators, nurse educators, health coaches, dieticians, etc. may help facilitate transformations in the social environment, and this change may be artificial or natural.^24^ Figure 2 presents constructs in sustenance of health behavior change in MTM.

Research suggests that public health and health promotion interventions that employ theoretical models rooted in the social and behavioral sciences are more effective than a theoretical interventions.^25,26^ However, theories should be empirically tested prior to being utilized for intervention development, implementation, or evaluation.^27^ Therefore, the purpose of this study was to test the utility of the MTM in predicting initiation and sustenance of small portion size consumption among college students. This study offers theoretical evidence regarding the efficacy of the MTM, which will guide the development of healthy portion size interventions targeting college students.

## Materials and Methods

### Study design, population and sampling

The present study utilized a cross-sectional design. The population for the study was college students, more specifically college students at a large size Southern public University in United States. The G*Power sample size calculation for regression modeling showed that a minimum of 114 participants were required to achieve a statistical power of 0.80 at an alpha level of 0.05 with 0.10 (medium) effect size and three predictors in the equation.^28^ This sample size was inflated by 15% for any missing values to arrive at a sample size of 131. While not ideal for structure equation modeling needed for confirmatory factor analysis, previous Monte Carlo studies suggest that this sample size was sufficiently powered to evaluate the hypothesized measurement models.^29^ This study utilized online quota sampling procedures. Participants were eligible to participate in this study if they were undergraduate or graduate students over the age of 18 who self-reported consuming a large portion size at a meal within the past 24 hours. The independent variables were constructs of MTM and the dependent variables were intention to initiate behavior change of eating small portion size and intention of continuing to eat small portion sizes.

### Instrumentation

The instrument consisted of 35 items, of which, seven asked respondents about their standard socio-demographic information (gender, age, ethnicity, class level, current grade point average, location of living, and work status). An additional 28 items measured the following MTM constructs for the two models:

### Initiation model

Advantages (participatory dialogue) were measured with five items (i.e., if you consume a small portion size at every meal you will… “be healthy,” “feel relaxed,” “manage your weight,” “have more energy,” and “enjoy life more”). Each item was scored on a five-point scale (0 = never to 4 = always). Responses for individual items were added together for maximum possible score (ranging from 0–20).

Disadvantages (participatory dialogue) were measured with five items (i.e., if you consume a small portion size at every meal you will … “feel tired,” “be hungry most of the time,” “have less energy,” “get sick,” and “have less enjoyment”). Each item was scored on a five-point scale (0 = never to 4 = always). Responses for individual items were added together for maximum possible score (ranging from 0–20).

In order to achieve the score on participatory dialogue (ranging from -20 – + 20), the total possible score of disadvantages was subtracted from the total possible score of advantages.

Behavioral confidence was assessed using five items. Participants were asked about their level of certainty to consume a small portion size in every meal “this week,” “this week while finding time to complete all academic/work-related task,” “this week while finding time for leisure,” “this week without feeling tired,” and “this week without feeling hungry.” Each item was scored on a five-point scale (0 = not at all sure to 4 = completely sure). Responses for individual items were added together for maximum possible score (ranging from 0–20).

Changes in physical environment was assessed using two items that asked participants about their level of certainty to “be able to eat a small portion size at a restaurant” and “be able to refuse a large portion size at a meal.” Each item was scored on a five-point scale (0 = not at all sure to 4 = completely sure). Responses for individual items were added together for maximum possible score (ranging from 0–8).

To measure initiation, participants were asked “how likely is it that you will eat small portion sizes at every meal in the upcoming week?” This item was followed by five-point response scale (not at all likely = 0 to completely likely = 4).

### Sustenance model 

Emotional transformation was assessed using three items that asked participants about their level of certainty of “directing feelings/emotions,” “motivating themselves,” and “overcoming self-doubt” to eat small portion sizes at every meal. Each item was scored on a five-point scale (0 = not at all sure to 4 = completely sure). Responses for individual items were added together for maximum possible score (ranging from 0–12).

Practice for change was assessed using three items that asked participants about their level of surety of “keeping a self-diary to monitor eating small portion sizes at every meal,” “be able to eat small portion sizes at every meal if you encounter barriers,” and “change your plan for eating small portion sizes at every meal if you face difficulties.” Each item was scored on a five-point scale (0 = not at all sure to 4 = completely sure). Responses for individual items were added together for maximum possible score (ranging from 0–12).

Changes in social environment was assessed using three items that asked participants about their level of surety of asking help from “family member,” “friend,” and “health professional” to support you eating small portion sizes at every meal. Each item was scored on a five-point scale (0 = not at all sure to 4 = completely sure). Responses for individual items were added together for maximum possible score (ranging from 0–12).

To measure sustenance, participants were asked “how likely is it that you will eat small portion sizes at every meal from now on?” This item was followed by five-point response scale (not at all likely = 0 to completely likely = 4).

### Face and content validity 

A total of six experts in the area of health behavior research were selected from multiple institutions to establish face and content validity of the instrument. Experts were requested to provide qualitative evaluation of the items. Based on experts’ recommendations, instrument was revised over a two-round process. The Flesch Kincaid Reading Ease of the instrument was 47.4 and Flesch-Kincaid Grade level of the instrument was 8.5 and thus acceptable for administration to college students.^30^

### Construct validity

In order to determine the factor structure, we performed a confirmatory factor analysis (CFA) in which we analyzed covariance matrices applying maximum-likelihood estimation using Mplus version 7.28. We used four indices to determine how well our models fit the data:^31^ chi-square, root mean square error of approximation (RMSEA), comparative fit index (CFI), and standardized root mean square residual (SRMR). RMSEA values of 0.06 or less, in conjunction with CFI values of 0.95 or greater were considered indicative of good fit. Models were considered to have adequate fit if they met the less stringent, but traditionally accepted, values of 0.90 or greater for CFI, and values less than 0.08 for RMSEA. We also included SRMR because it has been identified as the index that is most sensitive to miss-specified factor covariances or latent structures. For SRMR, values less than 0.10 are acceptable, with values less than 0.08 being preferred.

### Reliability

Cronbach’s alpha was calculated to determine internal consistency reliability. The results of Cronbach’s alpha for all subscales are reported in the Results section.

### Data analyses

Descriptive statistics were performed on all study variables. Data were checked for the assumptions of multiple regression (i.e., homoscedasticity, existence, independence, linearity, and normality). Stepwise multiple regression modeling for initiation and sustenance were conducted separately to determine best possible predictors of portion size behavior change while controlling for the socio-demographic variables namely age, race, and gender. For stepwise multiple regression procedure, the *a priori* probability levels for F to enter the predictor in the model and F to remove the predictor from the model were chosen as ≤ 0.05 and ≥ 0.10, respectively. All statistical analyses were conducted using SPSS (version 20.0). For gender the reference category was females and for race the reference category was other races as compared to Whites.

## Results

A total of 135 participants completed this study. The mean age of the study sample was 23.27 (SD: 6.11) years. The majority of participants (62.2%) were women. Whites represented 77.8% of the sample. Participants’ characteristics are presented in Table 1.

**Figure 3:**
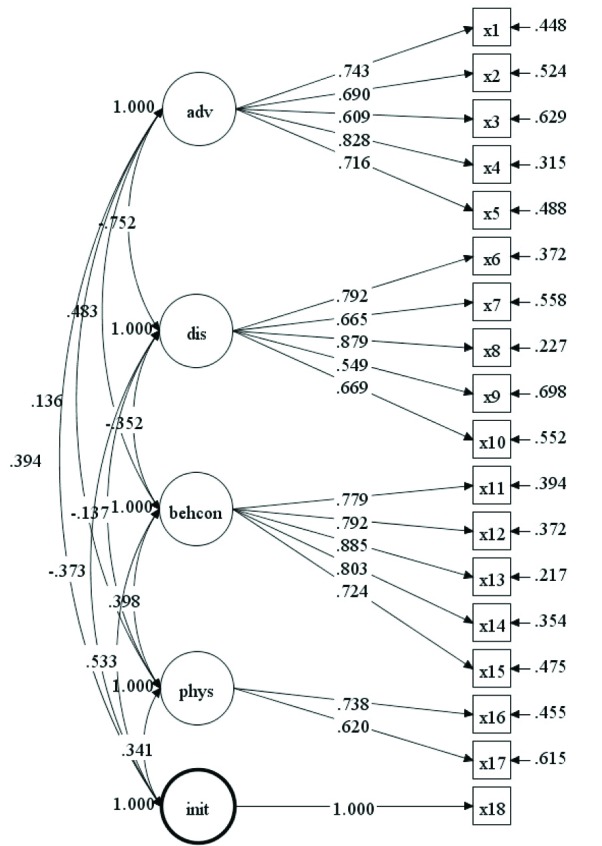
Confirmatory factor analysis for initiation model.
Abbreviations: adv, advantages; dis, disadvantages; behcon, behavioral confidence; phys, changes in physical environment; init, initiation. All item loadings are significant to *P*<0.001.

The path diagram in Figure 3 presents the findings for the CFA in Model 1. Fit for the model was good: χ^2^=239.40 (df=126), *P*<0.001, χ^2^/df=1.90, CFI=0.90, RMSEA=0.08 (90% CI=0.07-0.10), SRMR=0.06. Additionally, all item loadings were significant at *P*<0.001. Latent covariances ranged from −0.75 between advantages and disadvantages, to 0.53 between initiation and behavioral confidence. Chi-square difference tests showed that an alternative one-factor model achieved poorer fit (χ^2^=625.29 (df=135), *P*<0.001, CFI=0.58, RMSEA=0.16, SRMR=0.12).

**Figure 4:**
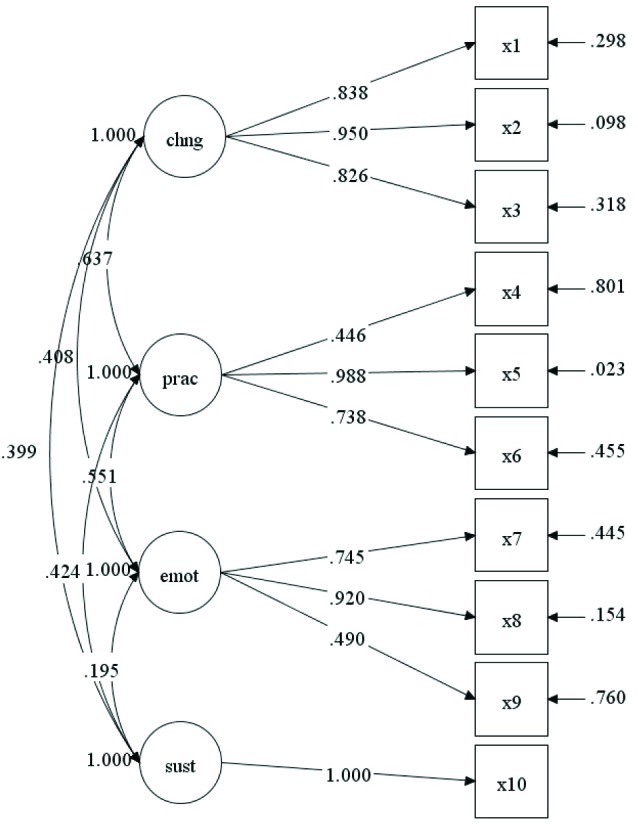
Confirmatory factor analysis for sustenance model.
emot, emotional transformation; prac, practice for change; chng, changes in social environment; sus, sustenance. All item loadings are significant to *P*<0.001.

The path diagram in Figure 4 presents the findings for the CFA in Model 2. Fit for the model was good: χ^2^=47.51 (df=30), *P*<.05, χ^2^/df=1.58, CFI=0.97, RMSEA=0.07 (90% CI=0.25-0.10), SRMR=0.04. Additionally, all item loadings were significant at *P*<0.001. Latent covariances ranged from 0.20 between emotional transformation and sustenance, to 0.64 between practice for change and changes in social environment. Chi-square difference tests showed that an alternative one-factor model achieved poorer fit: χ^2^=243.60 (df=35), *P*<0.001, χ^2^/df=6.96, CFI=0.68, RMSEA=0.21 (90% CI=0.19-0.24), SRMR=0.12. In sum, the analyses for both models support the hypothesized factor structure of the variables.

Table 2 presents reliability coefficient of the subscales and the scale as a whole. As shown in Table 2, the Cronbach’s alpha for all subscales were over 0.70 except for the subscale on physical environment which was close to 0.65. In behavioral and social sciences, scales with Cronbach’s alpha greater than 0.70 are considered respectable and those around 0.65 are considered minimally acceptable.

From Table 2 it is also evident that the mean score for the construct advantages was 10.25 units (SD: 3.71) which indicated that the participants’ sometimes view eating small portion sizes as beneficial. For the disadvantages construct, the mean score was 9.31 units (SD: 3.73) which showed that participants’ sometimes view eating small portion sizes as disadvantageous. With regard to behavioral confidence, the mean of 5.25 units (SD: 4.79, median 5, range 0-20) indicated that the participants were less sure to eat small portion sizes. The mean score for changes in physical environment was 2.47 units (SD: 1.95) which demonstrated that participants were less sure to make changes in physical environment to eat small portion sizes. Finally, the participants had a mean of 1.01 units (SD: 1.08) which represented that participants were less likely to eat small portion sizes at every meal in the upcoming week.

For the construct of emotional transformation, the mean score was 4.28 units (SD: 3.47) which indicated that participants were less sure in converting their emotions toward engagement in eating small portion sizes. The mean score for the practice for change construct was 3.68 units (SD: 2.86) which showed that participants were less sure to prepare themselves to eat small portion sizes. With regard to changes in social environment, the mean of 3.68 units (SD: 2.86) indicated that participants were less likely to take help of family member or friend to eat small portion sizes. Finally, the participants had a mean of 0.63 units (SD: 0.95) which represented that participants were less likely to eat small portion sizes at every meal from now on.

Table 3 depicts the results of stepwise multiple regression analysis for initiation model. It indicated that 37.1% of the variance in the initiation of small portion size consumption was explained by participatory dialogue (advantages outweighing disadvantages), behavioral confidence, age, and gender, F (4, 130) = 20.773, adjusted R^2^= 0.37, *P*<0.001. For gender, males were less likely to initiate small portion size consumption than females. The construct of physical environment was not significant.

Table 4 depicts the results of stepwise multiple regression analysis for sustenance model. It indicated that 20.5% of the variance in the sustenance of small portion size consumption was explained by emotional transformation, changes in social environment, and race, F (3, 131) = 12.535, adjusted R^2^= 0.20, *P*<0.001. For race, Whites were less likely to sustain small portion size change than other races. The construct of practice for change was not significant.

## Discussion

The purpose of this article was to use MTM of health behavior change to predict small portion size consumption in college students. The study found that for intention to initiate small portion size consumption the significant predictors were participatory dialogue (advantages outweighing disadvantages), behavioral confidence, age, and being female. Participatory dialogue that underscores the advantages outweighing the disadvantages and behavioral confidence have been found to be beneficial in other behaviors as well such as physical activity behavior in college students.^32^ A variant of behavioral confidence has also been used by Poelman and colleagues in an intervention aimed at altering portion control behavior.^9^ While no studies have been conducted on portion size and age, it seems logical to propose as age increases, weight increases, and people would be more inclined to employ weight management strategies such as initiating portion size control. Likewise, this study found that women were more likely to initiate small portion size consumption. This also makes intuitive sense as women are generally more diet conscious and likely to engage in reducing their portion sizes. Further, Gans and colleagues found that portion sizes of Black women were large for most food items and were keen to reduce those.^33^

In this study the construct of physical environment from MTM was not found to be significant in initiation of small portion size behavior. It seems the role of physical environment is limited. For example, even if a person is served a large portion size he or she has the choice to leave the food, thereby diminishing the role of physical environment on this behavior. On the whole, the initiation model predicted 37.1% variance in the intention to initiate small portion size consumption which is substantial for behavioral and social science studies.

For intention to sustain small portion size consumption, this study found that it was explained by emotional transformation, changes in social environment, and race, F (3, 131)=12.535, *P*<0.001. For race, Whites were less likely to sustain small portion size change than other races. This study showed that being White decreases the chances of sustaining the intention of small portion size consumption. The constructs of emotional transformation and changes in social environment were significant; these constructs have been found to be significant in other behaviors as well such as physical activity behavior in college students.^32^ The construct of practice for change was not found to be significant in this study. This could be due to the fact that perhaps the respondents felt that keeping a diary to monitor portion sizes was too cumbersome. On the whole, the sustenance model predicted 20.5% variance in the intention to sustain small portion size consumption which is moderately substantial for behavioral and social science studies. Thus, the MTM appears to be a useful model for explaining both the initiation and sustenance of behavior change to small portion size consumption, and may be used in designing and evaluating health promotion interventions. Regression analyses also show that the constructs do not have much shared variance; hence, the constructs are independent of each other and are mutually exclusive, providing support for the application of the MTM to other health behaviors.

The participants reported very low intention to initiate small portion size consumption behavior change (mean of 1.01 [SD: 1.08] units) and low intention to sustain change for small portion size consumption (mean 0.63 [SD: 0.95] units). This finding underscores the need for developing interventions to promote small portion size consumption in this target population. Also evident from the low scores is that such interventions may be difficult as the motivation to change in the target population is very low in this regard. However, MTM offers a robust framework to design such interventions.

### Limitations

First, this research had a cross-sectional design which looks at all the variables at one time. As a result, temporality of association of variables cannot be established. Therefore, we cannot say that the MTM constructs occur before the portion size behavior. However, all the previous theories have indicated that the attitudinal and environmental constructs like the ones in MTM precede the behavior; consequently, we can also assume the same for portion size behavior in college students. Future studies need to utilize more robust study designs.

Second, the real behavior has not been measured in this study; intention for initiation of behavior change and sustenance of behavior change served as proxies for the behavior, which can be considered a limitation of this study. However, previous theories, particularly theory of reasoned action and theory of planned behavior have used intentions as proxies for behavior and shown that intentions precede behavior.^30^ Hence, the operationalization of behavior the way it was done in this study is justified. Future studies can operationalize behavior more objectively.

Third, the instrument utilized in this study was based on self-report which is subject to measurement bias. Self-report, especially when it comes to assessing one’s portion size, can lead to recall bias, dishonesty, false reporting, under reporting, extreme reporting and other biases. However, there are no other methods to assess attitudes, therefore, this limitation must be considered within that context.

Fourth, since this was a convenience quota sample the results for this study are only applicable to this sample and strictly speaking cannot be generalized or are not externally valid. However, sample size estimation was performed and the purpose of the study was model testing for which the methods were appropriate.

Finally, the test-retest (stability) reliability of the instrument was not done in this study. Hence, it cannot be concluded that the constructs measured in this study are indeed reliable over time. Test-retest reliability assessment should be mandatory for replication studies.

### Implications for practice

It is clear from this study that there is a definitive need for designing and evaluating interventions to change portion size consumption behavior in college students. MTM offers a robust framework to design such interventions and evaluate them for efficacy and effectiveness. Such interventions can consist of one-on-one counseling, group interventions or campus wide campaigns. In order to impact initiation of small portion size consumption behavior the two constructs that this study found to be significant were participatory dialogue, which underscores the importance of advantages exceeding disadvantages, and behavioral confidence. Participatory dialogue is easy to implement in one-on-one counseling and group interventions where the facilitator (i.e., counselor, health educator, health education specialist, physician, other health care provider) can promote an open, two-way discussion of the advantages and disadvantages of the behavior, and swing the discussion in favor of the advantages. In a campus wide campaign, one would need to be innovative with regard to participatory dialogue where use of social media (i.e., Facebook, Twitter etc.) or emails may have to be employed in facilitating a two-way dialogue with a large audience. To build behavioral confidence, the behavior of small portion size consumption could be broken down into small steps, confidence may be built to perform the behavior in near future, and the person’s motivation be strengthened to reduce the portion size. This can be accomplished at the individual level through one-on-one counseling and at the group level by group discussion or other affective strategies such as role play. At the campus level, practices such as psychodrama can be utilized.

In order to impact sustenance of small portion size consumption behavior, emotional transformation and changes in social environment should be targeted. For modifying emotional transformation, the participants should be trained to direct their emotions such as anger, frustration, anxiety, etc. toward a goal of consuming small portion sizes. The skills to continually self-motivate oneself and overcome self-doubt in achieving this goal must also be taught. This may be accomplished through one-on-one counseling or group dialogue, or for campus wide campaigns, in the form of campus-wide contests or interaction via social media. Finally, in order to influence the construct of social environment, support from family, friends, and health professionals should be mobilized for interventions at all three levels.

## Acknowledgements

We would like to thank all the participants who participated in this research study.

## Ethical approval

This research study was approved by the University Institutional Review Board (IRB). After IRB approval, an online questionnaire was sent out to students who had been enrolled in the spring semester of 2016. All research participants provided informed consent electronically. The data for the present study were collected over a three-week period. Two reminder emails were sent to students in the second and third week.

## Competing interests

None to declare.

## Authors’ contributions

Manuscript conceptualization: MS, HPC, VL, and VKN; Manuscript writing: MS, HPC, VKN, VL, PJ, and MAF; Literature review: HPC and VL; Instrument development: MS; Data collection: VKN and MAF; Data analysis: VKN, MS, and PJ; Data interpretation: MS, VKN, HPC, VL, PJ, and MAF.

**Table 1:** Socio-demographic characteristics of the participants (n = 135)

	**Summary statistics**
Age (years)	23.27 (6.11)
Gender	
Male	51 (37.8%)
Female	84 (62.2%)
Race/Ethnicity	
White/Caucasian	105 (77.8%)
African American	12 (8.9%)
Asian American	7 (5.2%)
American Indian	2 (1.5%)
Hispanic American	2 (1.5%)
Other	7 (5.2%)
Class level	
Freshmen	21 (15.6%)
Sophomore	24 (17.8%)
Junior	25 (18.5%)
Senior	26 (19.3%)
Graduate	39 (28.9%)
Current overall GPA	
Less than 1.99	1 (0.7%)
2.00–2.49	5 (3.7%)
2.50–2.99	20 (14.8%)
3.00–3.49	40 (29.6%)
3.50–4.00	69 (51.1%)
Living arrangements	
On campus	36 (26.7%)
Off-campus	99 (73.3%)
Work Status	
Yes	72 (53.3%)
No	63 (46.7%)

Mean (SD) is presented for age and n(%) for other variables.

**Table 2:** Descriptive statistics of study variables (n=135)

**Constructs**	**Possible Range**	**Observed Range**	**Mean (SD)**	**Cronbach’s alpha**
Initiation	0–4	0–4	1.01 (1.08)	–
Participatory dialogue: advantages	0–20	0–20	10.25 (3.71)	0.84
Participatory dialogue: disadvantages	0–20	0–20	9.31 (3.73)	0.84
Participatory dialogue: advantages – disadvantages score	-20 – +20	-20 – +17	0.94 (6.68)	–
Behavioral confidence	0–20	0–20	5.25 (4.79)	0.90
Changes in physical environment	0–8	0–8	2.47 (1.95)	0.63
Sustenance	0–4	0–4	0.63 (0.95)	–
Emotional transformation	0–12	0–12	4.28 (3.47)	0.90
Practice for change	0–12	0–12	3.68 (2.86)	0.73
Changes in social environment	0–12	0–12	3.68 (2.86)	0.76
Entire scale	–	–	–	0.81

**Table 3:** Parameter estimates based on stepwise regression analysis to predict initiation of portion size consumption behavior change (n = 135)

**Variables**	**B**	**SE**_B_	**β**	**95% CI**	***P *** **value**
Participatory dialogue (advantages outweighing disadvantages)	0.035	0.012	0.214	0.010–0.059	0.006
Behavioral confidence	0.101	0.017	0.447	0.067–0.135	<0.001
Age	0.035	0.012	0.200	0.011–0.060	0.005
Gender (males)	-0.411	0.153	-0.185	-0.714 – -0.108	0.008

*F*(4, 130) = 20.773, *P* < 0.001, R^2^(Adjusted R^2^) = 0.390 (0.371). Dependent variable is initiation of physical activity behavior change; B = unstandardized coefficient; SE_B_ = standard error of the coefficient; β = standardized coefficient; *P* = level of significance; CI = confidence interval.

**Table 4:** Parameter estimates based on stepwise regression analysis to predict sustenance of portion size consumption behavior change (n=135)

**Variables**	**B**	**SE**_B_	**β**	**95% CI**	***P*** ** value**
Emotional transformation	0.074	0.022	0.272	0.030–0.119	0.001
Changes in social environment	0.050	0.023	0.174	0.004–0.096	0.033
Race (Whites)	-0.614	0.178	-0.269	-0.967 – -0.261	0.001

*F*(3, 131) = 12.535, *P* < 0.001, R^2^(Adjusted R^2^) = 0.223 (0.205). Dependent variable is sustenance of physical activity behavior change; B = unstandardized coefficient; SE_B_= standard error of the coefficient; β = standardized coefficient; *P* = level of significance; CI = confidence interval.
